# Retinoic acid receptor structures: the journey from single domains to full-length complex

**DOI:** 10.1530/JME-22-0113

**Published:** 2022-10-11

**Authors:** Fraydoon Rastinejad

**Affiliations:** 1 Nuffield Department of Medicine, University of Oxford, Target Discovery Institute (NDM RB), Oxford, UK

**Keywords:** structure, retinoic acid, receptors, hormone receptors

## Abstract

The retinoic acid receptors (RARα, β, and γ) are multi-domain polypeptides that heterodimerize with retinoid X receptors (RXRα, β, and γ) to form functional transcription factors. Understanding the three-dimensional molecular organization of these nuclear receptors (NRs) began with RAR and RXR DNA-binding domains (DBDs), and were followed with studies on isolated ligand-binding domains (LBDs). The more complete picture emerged in 2017 with the multi-domain crystal structure of RXRα–RARβ on its response element with retinoic acid molecules and coactivator segments on both proteins. The analysis of that structure and its complementary studies have clarified the direct communication pathways within RXR–RAR polypeptides, through which DNA binding, protein–ligand, and protein–protein interactions are integrated for overall functional responses. Understanding the molecular connections in the RXR–RAR complex has benefited from direct observations of the multi-domain structures of RXRα–PPARγ, RXRα–LXRβ, HNF-4α homodimer, and androgen receptor homodimer, each bound to its response element. These comprehensive NR structures show unique quaternary architectures, yet all have DBD–DBD, LBD–LBD, and DBD–LBD domain–domain contacts within them. These convergence zones allow signals from discrete domains of their polypeptides to be propagated and integrated across their entire complex, shaping their overall responses in an allosteric fashion.

## Introduction

**Figure 1:**
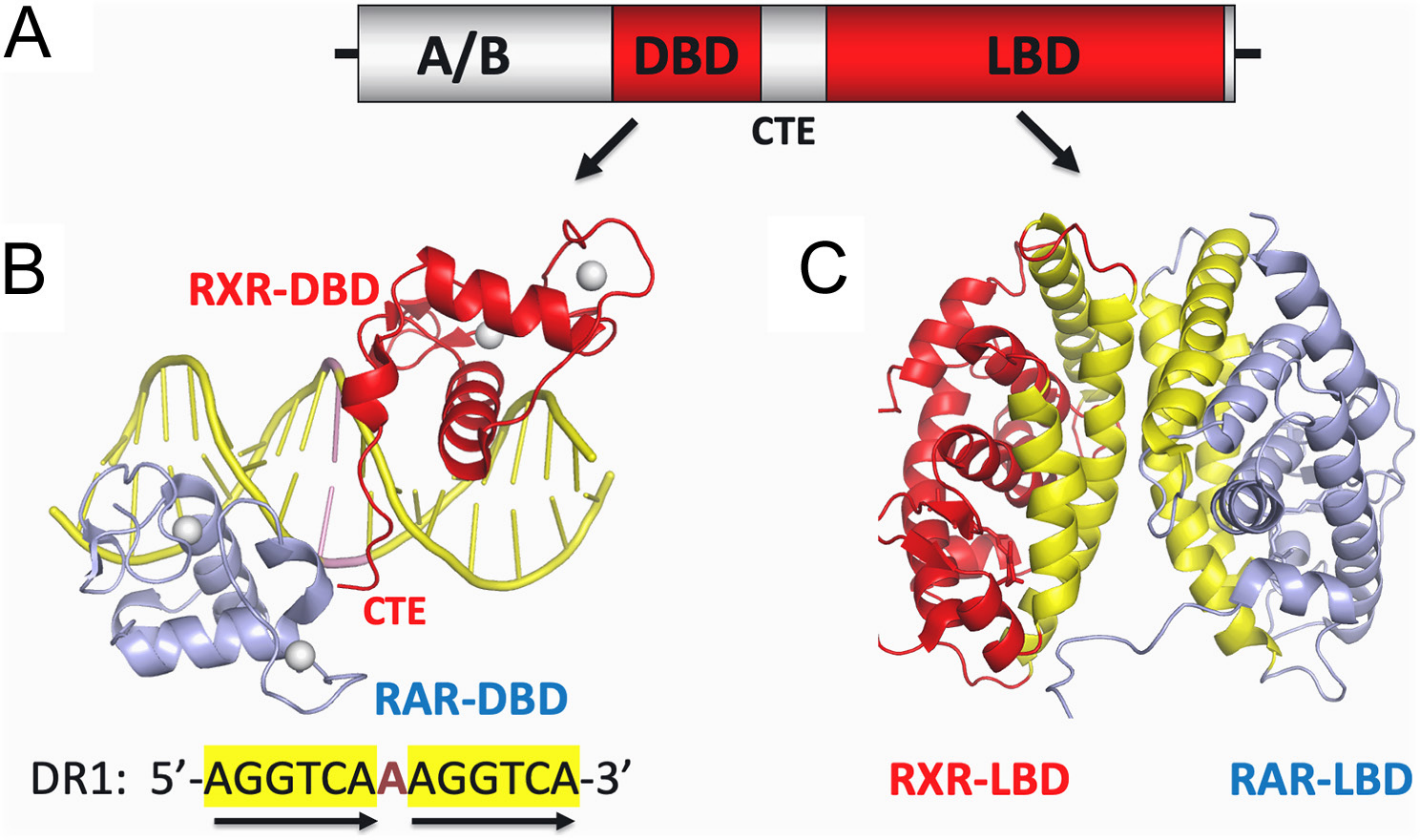
Individual domains of RXR–RAR polypeptides and their abilities to form DBD–DBD and LBD–LBD interactions. (A) The generalized polypeptide organization of NRs showing domain locations. (B) The first crystal structure for RXR–RAR, observed on a complex with its DR1 response element (Rastinejad *et al.* 2000) PDB 1DSZ. RARα binds to the 5’-AGGTCA half-site and RXRα binds to the 3’ half-site of the DR1 element. Each DBD uses its recognition helix to recognize half-sites at their major grooves. In addition, there is an observed DBD–DBD interaction that is DNA dependent, forming at the one base-pair spacer minor groove of the DR1 element. (C) The crystal structure of the RXRα–RARβ LBD–LBD dimer (Pogenberg *et al.* 2005) PDB 1XDK. The dimerization interface that joins two LBDs is DNA independent, and much larger in size than that formed between the two DBDs, in terms of buried solvent exposed surfaces. The LBD–LBD interface involves helices H7, H9 and H10, and loops L8–9 and L9–10 of each subunit (shown in yellow) which face each other.

The retinoic acid receptors (RARs) and retinoid X receptors (RXRs) are the earliest and most intensely studied nuclear receptors (NRs) for their three-dimensional (3D) structures. Both receptor groups bind to retinoic acids (RAs) to manifest their physiological effects through genetic responses during development and adult states. RXRs and RARs cooperate physically at the protein level by forming a heterodimer that allows response element binding and gene target specificity. The RAR and RXR polypeptides contain variable N-terminal domains (NTDs), centrally positioned DNA-binding domains (DBDs), and C-terminal positioned ligand-binding domains (LBDs), with DBDs and LBDs being conserved and well-folded segments whose architectures are known to be shared across the entire NR family (Fig. 1) (Khorasanizadeh & Rastinejad 2001, Huang *et al.* 2010, Rastinejad *et al.* 2013).

Three separate RAR genes that encode the NRs RARα, RARβ, and RARγ are conserved across vertebrates, with some having tissue-restricted expression patterns (Giguere *et al.* 1987, Benbrook *et al.* 1988, Zelent *et al.* 1989). Each RAR requires heterodimer formation with an RXR, and there are three RXR gene products (RXRα, RXRβ, and RXRγ) (Petkovich *et al.* 1987, Benbrook *et al.* 1988, Brand *et al.* 1988, Ragsdale *et al.* 1989, Evans & Mangelsdorf 2014). The RARs can bind all-trans RA or 9-*cis* RA, and RXRs bind to *9-cis* RA isomer (Huang *et al.* 2014).

The DBDs of the RXR and RAR are responsible for their DNA response element specificity (Kliewer *et al.* 1992, Mangelsdorf & Evans 1995, Evans & Mangelsdorf 2014, Rastinejad 2001). Ligand binding to RXRs and RARs follows a more general concept appreciated for the NR family by which endogenous lipophilic molecules, representing cellular signals, including RAs, vitamin D3, thyroid hormone, fatty acids and heme, are selectively bound in the hydrophobic pockets of LBDs (Huang *et al.* 2010). The LBDs further recruit coregulatory proteins in a ligand-dependent fashion (McKenna *et al.* 1999, Glass & Rosenfeld 2000, Lonard *et al.* 2007).

Additionally, the RXRs occupy a privileged position of being the required heterodimerization partners to many other NRs, including thyroid hormone receptor (TR), peroxisome proliferator activated receptors (PPARs), liver X receptors (LXRs), vitamin D_3_ receptor (Evans & Mangelsdorf 2014). In some cases, ligands binding to RXR’s partner alone drive the physiological response of their heterodimer, and in other complexes, RXR ligand binding further contributes to gene regulation (Mangelsdorf & Evans 1995, Huang *et al.* 2014).

To understand how RARs and their complexes function at the molecular level, they were subjected to NMR and X-ray crystallographic studies beginning in the 1990s. The first structural reports were published in 1993–1994 and focused on the isolated DBDs of RAR and RXR (Knegtel *et al.* 1993, Lee *et al.* 1994). That was followed by reports on isolated LBDs of RXRα, RXRβ, and RARγ (Bourguet *et al.* 1995, Renaud *et al.* 1995). Over these years, the LBD structural reports multiplied substantially in number, and other NRs also had their LBD structures determined in complexes with variety of distinct ligands and peptides (Huang *et al.* 2010). The ligands I emphasize in this review are RAs. Synthetic ligands and their actions through RARs and RXRs have been reviewed elsewhere (Huang *et al.* 2014).

From early cellular studies, it became clear that RXR–RAR heterodimers and other NR complexes displayed allosteric behaviors, wherein different ligands or response elements could influence the overall activation or repression profiles of their target genes (Kurokawa *et al.* 1994). However, the emphasis on determining isolated LBD structures could not yield a picture of how all the domains in the RXR–RAR heterodimer were interconnected to allow their overall integration. The structure for the multi-domain quaternary structure of RXR–RAR heterodimer bound to DNA, ligands, and coregulator peptides, together with a wide range of accompanying studies was reported in 2017, providing a detailed picture of how discrete domains within this complex are wired to communicate (Chandra *et al.* 2017).

Here, I look at how the molecular understanding of RARs and RXRs evolved from single-domain structures to culminate with understanding of its multi-domain heterodimeric complex at a level that accounts for allosteric responses to different signals and their functional integration. I focus mainly on results obtained from crystallographic, NMR, and hydrogen–deuterium exchange mass spectrometry (H/D-ex MS) where the details of the architecture and dynamics are clearly visible at resolutions that range from atomic level to protein side-chain level.

## DNA-binding domains

The DBD portions of RARs and RXR are responsible for several functions and have been subjected to intensive NMR and X-ray diffraction studies over the years (Rastinejad *et al.* 2013). NR DBDs typically recognize a hexameric half-site within their DNA response elements by interacting with the major grooves of its base pairs. They also form cooperative DBD–DBD interactions to help establish heterodimeric arrangements that give rise to a further ability to recognize the geometric features of their bipartite elements. A third, and more recently identified function, is their ability to contact the LBD portion in the context of full-length receptor, to allow for allosteric communication across their entire polypeptides (Chandra *et al.* 2017). This last feature is further discussed in the section on full-length receptors. Finally, the DBDs and their adjacent C-terminal amino acids can mediate the nuclear export of NRs (Black *et al.* 2001).

The first clues about DBD structures and their DNA-binding complexes came from NMR studies on the glucocorticoid receptor (GR) and estrogen receptor (ER) proteins (Hard *et al.* 1990, Schwabe *et al.* 1990, Knegtel *et al.* 1993). Those and subsequent crystallographic studies unveiled the secondary structure elements and the role of eight invariant cysteines within the DBDs of all NRs, responsible for coordinating two zinc atoms (Freedman *et al.* 1988, Luisi *et al.* 1991). The globular fold of the DBD contains two perpendicularly oriented α-helices, with one inserting in the major groove to recognize six base-pair half-sites. Given there are only two α-helices contained as their only secondary structure elements, DBDs can show a high degree of flexibility (Orlowski *et al.* 2004).

The steroid receptor members of the NR family, such as GR, progesterone receptor, androgen receptor (AR), and mineralocorticoid receptor, recognize the consensus 5′-AGGACA-3′ half-sites, while RXRs and partners including RAR heterodimers recognize 5′-AGGTCA-3′ half-sites (Rastinejad *et al.* 2013). Furthermore, the steroid receptors bind to palindromic DNA repeats containing two hexamers, whereas RXRs and its partners bind to direct repeats (DRs) (Umesono *et al.* 1991, Zechel *et al.* 1994, Rastinejad *et al.* 2013). Further specificity for DR elements comes from their variable half-site spacings, which can vary from one to five base pairs in size (i.e. DR1–DR5 elements) (Umesono *et al.* 1991, Mader *et al.* 1993). Detailed examinations of how half-site sequences interact with DBD recognition helices have shown flexible modes that include cases where water molecules can bridge some or all the contacts required for base-pair recognition (Sierk *et al.* 2001).

The different spacings of DR elements amount to restricted geometric demands on RXR heterodimers. Each additional intervening base pair introduces a 3.4 Å displacement and ∼35° degree rotation between the half-sites to be occupied by receptor pairs, in some cases making their cooperation possible and in other cases preventing their joint binding (Rastinejad *et al.* 1995*b*). The individual half-sites of DR elements can further be distinguished according to their upstream or downstream location when they are bound by a heterodimer like RXR–RAR. RXR can bind either to the upstream or downstream half-site relative to RAR. Indeed, RXR–RAR complexes on DR1 and DR5 are known to display opposite polarities and to opposing functional responses (Kurokawa *et al.* 1994). Accordingly, differing response elements can differentially configure RXR–RAR complexes to influence their functional outputs as transcriptional regulators.

The first RAR or RXR domain whose structures were reported were the isolated RARb DBD and RXRα DBD (Knegtel *et al.* 1993, Lee *et al.* 1994). Those NMR structures confirmed that RAR and RXR DBDs looked highly similar to GR and ER DBDs, as had been expected due to high sequence similarity. Yet the RXR DBD structure revealed an additional short helix at its C-terminal extension (CTE) (Lee *et al.* 1994). In the ensuing years, our appreciation for CTEs having functional roles in terms of DNA recognition and discrimination increased, as monomeric DBD/DNA complexes for other NRs began to be visualized crystallographically (Zhao *et al.* 1998, Meinke & Sigler 1999, Rastinejad *et al.* 2013).

Structural insights about how RXR and its partners use their DBDs to cooperatively recognize DR response elements came from the co-crystal structure for RXRα–TRα DBD complex on DR4 DNA (Rastinejad *et al.* 1995*b*). That first report showed how RXRs cooperated with partners such as the TR via their DBDs. Each DBD engaged its AGGTCA half-sites using its recognition helices. But the structure also showed a DNA-dependent DBD–DBD dimerization interface between them. Their interface was formed within the DR spacer element and along the minor groove. The asymmetrical head-to-tail placement of the RXR and TR DBDs on DR4 with RXR upstream of TR allowed their DBD–DBD dimerization surfaces to lock in. Moreover, the CTE portion in TR had a significant α-helical structure stabilized by DNA, and this helix functioned as a ruler that could discern the correct half-site spacing of DR4 and preclude the binding to DR elements with smaller spacings.

The first structural visualization of the DBDs of RXRα–RARα which involved their bound complex on a DR1 was published in 2000 and is shown in Fig. 1B (Rastinejad *et al.* 2000). RXR was on the downstream half-site, in contrast to its upstream position in the RXR–TR complex. The recognition helices of RAR and RXR directly engaged the major grooves of their hexameric half-sites. Again, the minor groove of the spacer element was the site where RXR and RAR formed their DBD–DBD interaction surface, suggesting that this dimerization interface was spacer DNA dependent.

Soon after, several other crystal structures were reported for RXR DBD and its partner DBDs on response elements, including structures of RXR ortholog in *Drosophila* known as ultraspiracle in complex with the ecdysone receptor (Zhao *et al.* 2000, Devarakonda *et al.* 2003). Other response elements for RXR–RAR besides DR1 and DR5 are believed to include IR0, DR0, DR2, and DR8 (Moutier *et al.* 2012). More recently, crystal structures of RXR–RAR DBD complexes were also reported on DR5 and DR0 elements (Osz *et al.* 2020).

## Ligand-binding domains

Like DBDs, the LBD portions of NRs conduct multiple functions. They contain specific pockets that allow the discrimination of their endogenous ligands. They have a so-called activation function (AF-2), which refers to their ability to use ligand binding to induce conformational changes that promote the exchange of coregulators (chromatin modifiers, coactivators, and corepressors) (Dasgupta *et al.* 2014, Giudici *et al.* 2016). In addition, LBDs can dimerize with each other in a DNA-independent manner. A side by side stereochemical description for ligand recognition by various NRs, covering their abilities to bind to endogenous ligands alongside drug-like molecules, has been found elsewhere (Rastinejad *et al.* 2013).

Coactivators and corepressor typically contain short peptide motifs within their polypeptides for binding to NR LBDs. In the case of coactivators, proteins such as steroid receptor coactivators (SRCs) contain three or more leucine-rich, LXXLL motifs within a single polypeptide (Heery *et al.* 1997, Darimont *et al.* 1998). In the case of corepressors, the LXXXLXXX (I/L) motif is used for binding to NR LBDs, and these motifs are also known as CoRNR boxes (Hu & Lazar 1999). The coactivators typically used by RARs and RXRs act as scaffolding surfaces for engaging with histone-modifying enzymes such as histone acetyltransferases or histone methyltransferases. Their enzymatic activities provide NRs access to their target DNA sequences within chromatin and can make those sites available to further regulatory proteins. Conversely, the unliganded RARs can engage with corepressor proteins and histone deacetylases that restrict their access to DNA elements in chromatin (Khorasanizadeh & Rastinejad 2016).

Crystal structures for LBDs of RXRα without ligand, and the ligand bound forms of RARγ-TRα dimers, were all reported in 1995 (Bourguet *et al.* 1995, Renaud *et al.* 1995, Wagner *et al.* 1995). By comparing the three LBDs, it was evident they shared a 3D arrangement consisting of 12 α helices. These helices together create a three-layered α-helical sandwich. These LBDs also harbored internal hydrophobic pockets for the ligand. Subsequent structural studies on other NR LBDs have confirmed that the 12-alpha helical fold is shared across the entire NR family (Huang *et al.* 2010).

The TRα and RARγ LBD structures reported in 1995 provided a structural explanation for ligand binding, since their physiological ligands were co-crystallized (Renaud *et al.* 1995, Wagner *et al.* 1995). It became clear that the amino acids in NR LBD pockets vary sufficiently to allow for ligand discrimination. Over the decades since those report, it has become evident that the sizes of NR ligand pockets can vary from nearly zero in volume (due to their occupancy by hydrophobic side chains) to >1500 Å^3^ (Huang *et al.* 2010). The pocket shapes and sizes are also recognized to be adaptive, enabling synthetic ligands with a wide range of functional groups to bind to LBD pockets.

The early structural studies on RARγ and RXRα LBDs led to an erroneous proposal of a mousetrap model in which the position of helix-12 would move between two discrete states upon binding and unbinding of ligand (Wurtz *et al.* 1996). I have pointed out previously how the mousetrap model was misguided since its authors failed to take into account a key crystal packing artifact that created an artificial positioning of helix-12 in the apo-RXR structure (Rastinejad *et al.* 2015). A dynamic stabilization model is now widely accepted to suitably describe how ligand binding actually induces a conformational change in LBDs where the switch between inactive and active states is a transition from disordered to ordered helical conformation of helix H12 (Nagy & Schwabe 2004).

Solution studies have supported this dynamic stabilization property upon ligand binding. Fluorescence and NMR studies have shown helix H12 is more dynamic in the apo-LBDs compared with the holo-LBDs (Kallenberger *et al.* 2003, Nolte *et al.* 1998, Johnson *et al.* 2000). In each of the full-length RXRα–RARβ and RXRα–PPARγ complexes, H/D-ex MS studies were used to examine the exchange rates of amide hydrogens along helix H12 (Chandra *et al.* 2008, 2017). The results were consistent with the dynamic stabilization model.

The LBD structures of all three isotypes of RAR and RXR have been determined over the years and reviewed alongside each other (Huang *et al.* 2014). RXR LBD pocket residues responsible for interacting directly with 9-*cis* RA were fully conserved in all three RXR isotypes. The available volume in the RXRα LBD pocket is nearly 500 Å^3^, but 9-*cis* RA occupies only 300 Å^3^, or 60% of the total available space. The high affinity of 9-*cis* RA for the RXR pocket is derived from favorable hydrophobic interactions of amino-acid side chains with the isoprene chain of the ligand (Huang *et al.* 2014). The role of the 9-*cis* RA isomer as the true endogenous RXR ligand has been challenged at times, since it is not detectable in many tissues (Mic *et al.* 2003, Kane 2012). By contrast, all-trans RA has been detected more widely in tissues, and its function as the endogenous ligand of RARs in all tissues is widely accepted.

9-cis RA is a high-affinity ligand for all three RXR subtypes (Heyman *et al.* 1992, Levin *et al.* 1992). A second endogenous RA isomer with potentially greater tissue availability is 9-cis-13,14-dihydroretinoic acid, and this molecule may be another RXR ligand (Ruhl *et al.* 2015). Another potential ligand is the unsaturated fatty acid docosahexaenoic acid (DHA), a major polyunsaturated fatty acid known to be enriched in mammalian brain in late gestation and early postnatal periods (de Urquiza *et al.* 2000). Furthermore, linoleic and linolenic acids and phytol metabolites can bind and activate RXRs (Wolf 2006). A detailed look at how fatty acids bind LBDs of RXRs shows that their mode of binding involves the entire chain and head groups becoming enveloped within the protein pocket, unlike other lipid-binding proteins where the fatty acid polar head groups remain solvent exposed (Wright *et al.* 2003, 2004, 2005, Huang *et al.* 2014, Diao *et al.* 2022). RXRs may employ several distinct types of lipophilic ligands based on their tissue availability and abundance. A more complete review of RARs and their structural interactions with candidate ligands can be found elsewhere (Huang *et al.* 2014).

**Figure 2:**
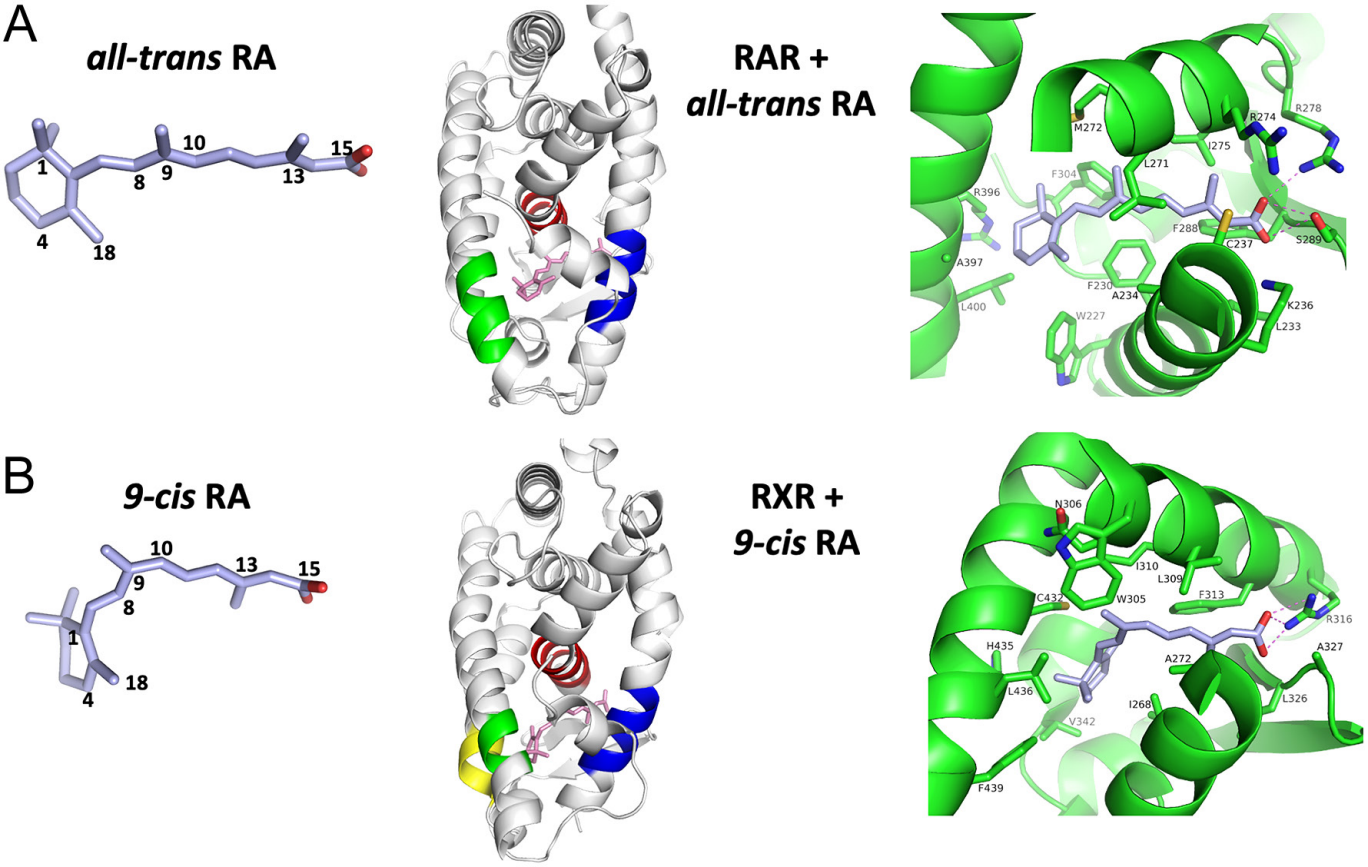
Molecular structures of RA molecules and their binding sites within RAR and RXR LBDs. (A) 3D structure of all-trans RA and its binding location and interactions with RARγ LBD (from PDB 2LBD). (B) 3D structure of *9-cis* RA and its binding location and interactions with RXRα LBD (from PDB 1FBY). Each ligand becomes lodged in a cavity formed mainly through helix-3 (blue), helix-5 (red) and helix-10/11 (green).

The comparison of RXR and RAR LBDs has explained ligand discrimination between RARs and RXRs. The ability of RXR to bind well to 9-*cis* RA, but not to all-trans RA, is explained by the shape of its LBD pocket (Egea *et al.* 2000). A sharp bend is responsible for the selective binding of 9-*cis* RA and is incompatible for all-trans RA binding (Fig. 2). The elongated and L-shaped binding pocket of RXR is sealed by an arginine of helix H5 on one end and helix H12 on the other end.

As shown in Fig. 2, 9-*cis* RA has a twist about the C8–C9 bond which orients the cyclohexenyl ring nearly perpendicular to the C9–C15 carbons, allowing binding to RXR LBD (Perez *et al.* 2012). RXR adapts to diverse ligands through displacement of amino-acid side chains within the pocket. As to the question of how a ligand activates the receptor by stabilizing helix H12, several hydrophobic residues of the pocket from helices H10/H11 and H3 communicate ligand presence to the AF-2 surface including H12. Indirect ligand communication with helix H12 through a distance has been commonly observed in other NR LBDs (Huang *et al.* 2010).

The LBDs are also involved in physically connecting RXR and RAR via a strong dimerization interface (Fig. 1C). More broadly, NR members can in some cases form heterodimers with RXRs and in other cases form their own homodimers or function as monomers (Rastinejad *et al.* 2013). In cases where they homodimerize or heterodimerize, the LBD–LBD buried surface is signifi­cantly larger that the DBD–DBD buried surface (Huang *et al.* 2010). The weaker nature of the DBD–DBD interface accounts for why its formation is exquisitely dependent on the participation of correctly spaced response elements. The LBD–LBD interfaces in contrast, through their more substantial interactions, are insensitive to whether DNA is present or not. LBD–LBD dimers were structurally examined using X-ray crystallography for RXRα–RARβ, RXRα–LXRβ, and RXRα–PPARγ. These interfaces were observed to involve helices H7, H9, and H10 and loops L8–9 and L9–10 (Bourguet *et al.* 2000, Gampe *et al.* 2000).

In each of those LBD–LBD heterodimeric structures, both subunits are observed to bind to their ligands and their coactivator-derived peptides independently, as had been repeatedly confirmed with subsequent multi-domain structures of NR heterodimers and homodimers. Helices H3–H5 together with the H12 portion of the LBD form the common coactivator NR–box–interaction surface which embodies the AF-2 function. This surface is largely exposed upon binding of activating ligands (agonists) including RA ligands in the case of RXRs and RARs. But the surface is blocked by inhibitory ligands (antagonists). Thereby, the conformational changes produced by agonists, such as that described in the dynamic stabilization model described above, shape the ability of coactivators to associate with the AF-2 surface on these LBDs (Huang *et al.* 2010). The physical connection formed between the LBDs of RXR–RAR in their heterodimers can allow for allosteric communication between these subunits. A so-called phantom ligand effect based on LBD to LBD allosteric communications was demonstrated early on (Schulman *et al.* 1997). Specifically, the binding of an RXR-specific ligand (LG100754) was found to cause the dissociation of corepressor and the association of coactivator on the adjacent RAR subunit, mimicking the effects one would expect when RAR itself is liganded.

## N-terminal A/B segments

In contrast to DBDs and LBDs, no stable structures or domains appear to be associated for the N-terminal segments of RARs and RXRs. This has been confirmed through our H/D-ex MS studies (Chandra *et al.* 2017). These segments showed rapid amide exchange rates consistent with lack of any stable secondary or tertiary structures. The A/B portions in some NRs contain a further transcription activation domain, called AF-1, whose function is independent of ligands. In some NRs, the AF-1 and AF-2 are known to cooperate in recruiting coactivators (Wärnmark *et al.* 2003, Yi *et al.* 2015), although this has not been explicitly shown in RARs. There is no evidence from the literature for ordered conformation in A/B regions of RARs and RXRs or their related NRs nor of their interactions with each other or any other portion of their polypeptides. This finding is consistent with a lack of sequence conservation in these segments and their low-complexity sequences. The region can contain phosphorylation sites, or other posttranslational regulatory sites, and this has been reported for one RAR subtype (Gianni *et al.* 2003). The phosphorylated AF-1 status in RARγ2 may partially drive the degradation of its receptor (Gianni *et al.* 2003). In the case of AR, the entire receptor polypeptides that included its NTDs were subjected to cryo-EM studies (Yu *et al.* 2020).

The AR has an AF-1 within this region that was studied in a cryo-EM structure that included the receptor homodimer, DNA, the SRC-3, and an additional coactivator p300. However, owing to the low resolution, the detailed structural features and interactions of this segment could not be discerned clearly at the atomic or amino-acid levels. It is known that the AR AF-1 is a key site for coactivator recruitment, and an FXXLF amino-acid motif within its N-terminal region is capable of interacting with the coactivator binding groove of its LBD (Dubbink *et al.* 2004). The cryo-EM structure showed that when the AR homodimer is assembled on DNA, the two N-terminal segments surround the LBD dimer so that only a small portion of the LBD is left exposed. Furthermore, the two N-terminal segments interact with the p300 protein (Yu *et al.* 2020). While these interactions involving the N-terminal region may be unique to AR and not necessarily common to other receptors such as RARs or RXRs, the findings make the compelling case that N-terminal segments can foster both intra- and intermolecular physical contacts within NR transcriptional complexes to control their functions.

## Full RXR–RAR structure and allosteric connections

**Figure 3:**
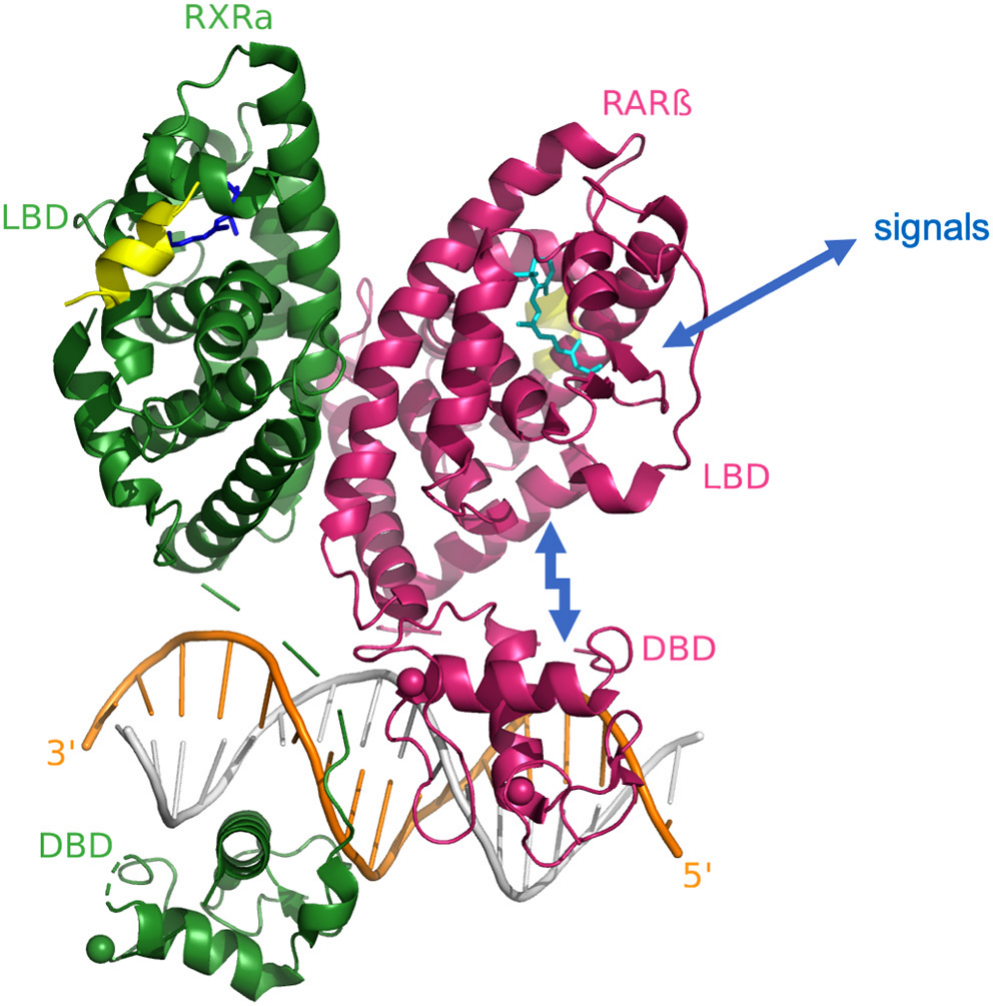
The multi-domain crystal structure of the RXRα–RARβ heterodimer with DNA, ligands, and coregulator peptides (Chandra *et al.* 2017) PDB 5UAN. All of the previously known LBD–LBD, DBD–DBD, and DBD–DNA interactions (Fig. 1) were preserved within this multi-domain complex. But there were new findings including the LBD–DBD interface within the RARβ protein. Biochemical and cell-based functional studies indicate that signals detected at the LBD (different ligands) or DBD (different response elements) can be allosterically transmitted in a bidirectional manner (Chandra *et al.* 2017).

The structural studies reliant on only DBDs or LBDs had left RXR–RAR allosteric properties difficult to explain. Different DR spacings on RXR–RAR response elements can lead to activation or repression, indicating that signals from the DBD are sent across the length of their polypeptides to distal portions including LBDs, where ligands and coregulators binding information integrate for gene regulation. Therefore, structural studies were needed on the multi-domain RXR–RAR heterodimer to reveal a clear wiring diagram involving multiple domains that underpins the allosteric properties of this complex (Fig. 3).

**Figure 4:**
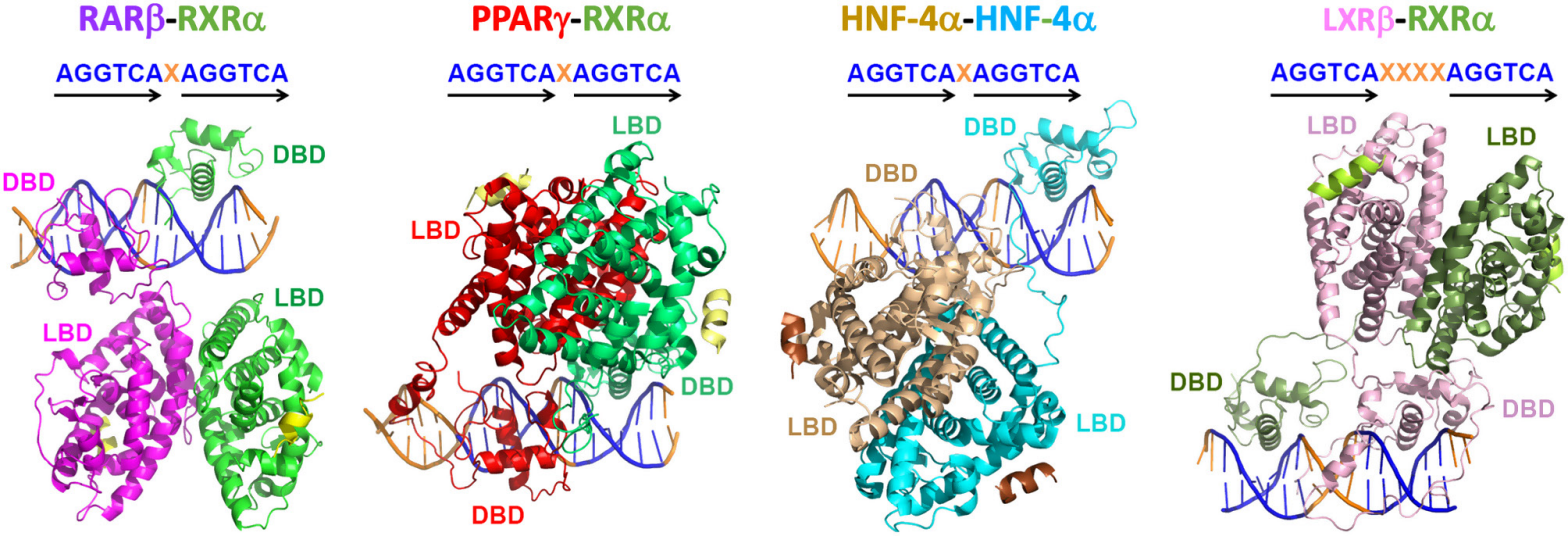
Uniqueness of NR quaternary structures. Here a comparison of the multi-domain quaternary crystal structures of RXRα–RARβ (Chandra *et al.* 2017) PDB 5UAN, RXRα–PPARγ (Chandra *et al.* 2008) PDB 3DZY, 3E00, 3DZU, HNF-4α homodimer (Chandra *et al.* 2013) PDB 4IQR, and RXRα–LXRβ–DR4 heterodimer (Lou *et al.* 2014) PDB 4NQA is shown. All four complexes are aligned the same way, with their DBDs and half-sites held in identical positions for direct comparison of quaternary structures.

However, a small number of multi-domain NR crystal structures with sufficient resolution and detail to see individual amino-acid side chains began to emerge beginning with our report on RXRα–PPARγ complex on DR1, in 2008 (Chandra *et al.* 2008). We subsequently reported the multi-domain structure of the hepatic nuclear factor-4α (HNF-4α) homodimer bound to DR1 in 2013 (Chandra *et al.* 2013), which was followed by the structure of multi-domain RXRα–LXRβ on DR4 in 2014 from the Gustafsson lab (Lou *et al.* 2014). We reported the crystal structure of multi-domain RXRα-RARβ on DR1 in 2017 (Lou *et al.* 2014). The DR complexes are all compared in Fig. 4. Another NR structure of comparable complexity subjected to studies delivering similar quality resolutions is the multi-domain AR on its DNA element from single-particle cryo-EM, which has recently provided its first detailed structural organization and described the allosteric behavior of that steroid receptor (Wasmuth *et al.* 2022).

**Figure 5:**
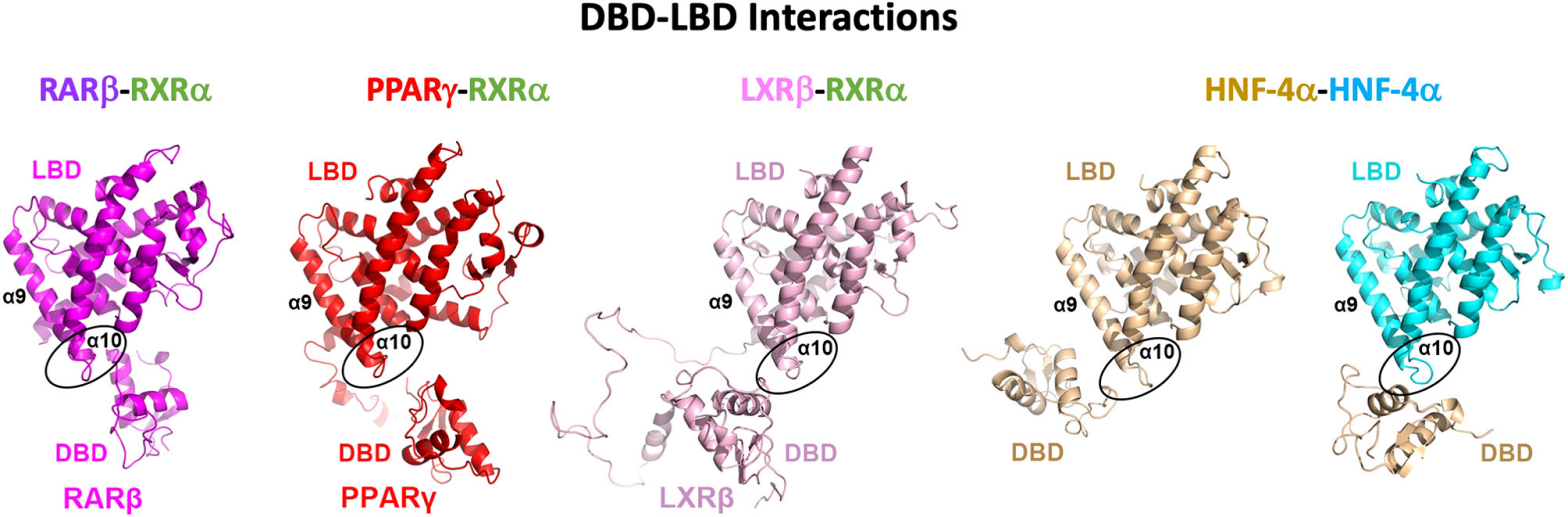
DBD–LBD interactions in all four NR–DNA complexes. The DBD–LBD interface is established between a loop in the DBD (preceding the DBD’s α1 helix) and the region of the LBD located between α9 and α10 helices The PDB IDs are indicated in Fig. 3.

To successfully subject these types of multi-domain heterodimeric complexes to high-resolution structural studies, their N-terminal A/B regions typically need to be removed based on their intrinsic disorder and high mobility. The addition of idealized DNA response elements, high-affinity ligands, and coregulator-derived peptides substantially stabilizes NR complexes, including their DBD and their hinge regions (located between DBD and LBD), enhancing likelihood of their crystallization. The four multi-domain crystal structures mentioned above showed unique quaternary structures (Fig. 4). Their variability can derive from their DNA elements and their differing subunit polarities on their response elements. Nevertheless, they all were found to have DBD–LBD domain–domain junctions (Fig. 5). In comparing these DBD–LBD interactions, it is clear that all complexes use the same region within their DBDs and a similarly positioned region from their LBDs (consisting of a region between helices H9 and H10) to create DBD–LBD interfaces (Fig. 5) (Chandra *et al.* 2017).

In the case of RXRα–RARβ, this multi-domain heterodimer (lacking A/B segments) was tested for binding to AGGTCA half-sites with one or five base-pair spacings, which in both cases showed high-affinity interactions with the DNA element. The RXRα–RARβ heterodimer with DR1 has been characterized at atomic detail in a complex containing 9-*cis* RA and all-trans RA and LXXLL synthetic peptides derived from its coactivator protein.

In the DR1 complex crystal structure, the domain–domain interfaces that were of greatest interest were clearly identified and subsequently confirmed through mutational studies. A striking feature was that the DBD and LBD domains of RARβ were in direct domain–domain contact. The corresponding two domains of RXRα, however, were distant from each other. Furthermore, there was no electron density for the connecting hinge region connecting those two domains, suggesting that the hinge region of RXR is flexible, which presumably is of importance for its ability to interact with many other heterodimeric partners and response elements.

At the same time, RXR–RAR’s LBDs and DBDs showed ligand and coactivator binding and a mode of LBD–LBD dimerization that was fully consistent with previous findings from single-domain structural studies (Fig. 3). Also the way in which the two DBDs cooperated to bind to DR1 DNA and the polarity of occupying DNA half-sites was exactly as previously described based on originally described RXR–RAR DBD–DNA crystal structures (Rastinejad *et al.* 2000). But the overall quaternary state of this heterodimer showed a number of exciting new architectural features and provided a blueprint for how discrete domains from each receptor contribute to the overall complex.

That structure was complemented by several solution-based studies. These studies confirmed the architectural arrangement and helped to further probe the allosteric behavior of the complex according to architecture. Antagonists and agonists of RARβ were tested on the heterodimeric complex, to see if switching between them produced conformational changes detectible by H/D-ex MS (Chandra *et al.* 2017). The ligand switching did clearly register a change, since the exchange rates observed along helix H12 were notably changed. Similarly, changing the DNA element from DR1 to DR5 clearly registered a change in the H/D-ex MS pattern seen at the DBD portions.

However, true allosteric communication between DBD and LBD domains still had to be probed in the RXRα–RARβ complex, given that the DBD–LBD interface was clearly visible in the RARβ protein (Fig. 3). Was this a convergence zone that allowed for propagation of information from one domain to another? There has been clear evidence for allosteric transmission from the LBD to the DBD in the case of multi-domain HNF-4α homodimer, based on using mutations introduced within the LBD (Chandra *et al.* 2013). But due to the nature of the non-exchangeable ligand in HNF-4α, it was not possible to assess the allosteric transmission of ligand type from the LBD to the DBD in that complex. The H/D-ex studies on RXRα-RARβ/DR1 showed that the RARβ DBD underwent a change in its H/D-ex MS pattern when an agonist was switched to an antagonist. Therefore, the DBD–LBD interface provided a sensitive route for allosteric transmission of signals between those domains.

The structure characterization of multi-domain RXRα-RARβ/DR1 has also seen an array of cell-based functional studies that further confirmed the structural inferences and also looked for integrated responses to ligands and response elements through the architecture. The importance of the RARβ DBD–LBD connection for transcriptional activity was tested on differing response elements (Chandra *et al.* 2017). Classic response elements from *ANGPTL4* (DR1) and *CYP26A1* (DR5) target genes of RXRα–RARβ were used in that study. RXR–RAR complexes were found to repress transcription from DR1, and that repression was further potentiated with the all-trans RA. On DR5, transcriptional activation was seen by RXRα–RARβ and that activation was further enhanced by *all-trans* retinoic acid (REA) addition.

Then RARβ DBD–LBD cross-coupling was tested in cell-based functional assays when mutations were introduced at that inter-domain junction (Chandra *et al.* 2017). Those point mutations abolished the transcriptional repression on DR1 and also abrogated the transcriptional activation from the DR5 reporter. This confirmed the importance of the DBD–LBD interface and also suggested that an RARβ DBD–LBD interface is likely to form on DR5 elements. Furthermore, a PKA phosphorylation site on RARα (S369) critical for modulating RARα-driven cell differentiation by all-trans RA (Rochette-Egly 2003) (corresponding to S362 in RARβ) localizes directly on the observed RAR DBD–LBD interface, further underscoring its role as a critical node for functional regulation.

H/D-ex MS studies on the full-length RXRα–PPARγ on DR1 has also provided a picture consistent with the crystallographically observed domain–domain interactions, its DBD–LBD interactions (Chandra *et al.* 2008). Since in the absence of their bound DNA, the DBD–DBD and DBD–LBD interactions are not stably maintained, an important role is played by the response element for stabilizing the overall quaternary organization. In the case of HNF-4α homodimer on DR1, it was shown that MODY1 mutations located on the LBD allosterically communicate to impact DNA-binding affinity at the DBD (Chandra *et al.* 2013), providing support that its DBD–LBD junction also is a sensitive nexus for signal communication between distal parts of its full-length complex.

There is also now clear evidence now in both the AR homodimer DNA complex and the ER homodimer DNA complex for DBD–LBD interactions (Huang *et al.* 2018, Wasmuth *et al.* 2022). The anti-androgen enzalutamide was known to allosterically inhibit AR’s ability to bind DNA (Wasmuth *et al.* 2020), consistent with subsequent structural observations of DBD–LBD contacts within the AR–DNA complex from cryo-EM studies (Wasmuth *et al.* 2022). Therefore, despite unique quaternary organizations in the NR–DNA complex, DBD–LBD physical contacts are likely common to steroid and non-steroid NR members, regardless of whether their response elements are palindromic or DRs. The full repertoire of domain–domain interfaces in each NR–DNA complex, including the DBD–LBD interactions, allows the actions of ligands to be manifested at multiple levels. These manifestations include the release of corepressors, the recruitment of coactivators, and modulation of DNA-binding affinity.

## Future directions

Going forward, the next level of desired structural information for RARs and RXRs concerns their intact complexes with coregulatory proteins. Our understanding of their N-terminal regions and their modes of interactions and functional outputs also needs expansion. Carefully conducted cryo-EM studies have begun to show how one can tackle such NR complexes in the case of steroid receptors, revealing the nature of their many domain–domain interfaces and including the characterization of their N-terminal interactions (Yi *et al.* 2015, Yu *et al.* 2020, Wasmuth *et al.* 2022). Extending cryo-EM studies and enhancing their resolution limits would generate significant new understanding about how signals can be propagated between various components of their functional complexes through physical couplings and allosteric communications. While to date crystallography has been able to provide most of the available high-resolution information about NR polypeptides, cryo-EM is more suitable for probing the larger and more disordered assemblies of interest which are unlikely to form crystals. The application of H/D ex MS to RAR and RXR receptor systems has already proved powerful in revealing ligand and DNA-induced conformational changes in these receptor proteins (Chandra *et al.* 2017). This methodology should continue to prove powerful in probing the details of allosteric changes within much larger NR complexes and their coregulators. Methods to observe the locally induced changes in DNA-binding element structure and dynamics upon protein binding using NMR have been available but have not yet been fully utilized for NR studies (Rastinejad *et al.* 1995*a*).

Transcription factors dominate the cancer vulnerability landscape, as shown by loss-of-function screens (McDonald *et al.* 2017, Tsherniak *et al.* 2017). The NR proteins have for decades stood out among transcription factors due to their tractable ligand-binding pockets, providing many opportunities for drug discovery. Most other transcription factors are not considered to have tractable pockets for drug targeting. However, a second major factor family, namely the bHLH-PAS proteins, has been recently recognized to contain conserved ligand-binding pockets, which also allow for ligands to control their activities in a bidirectional manner (Wu *et al.* 2015, 2016, 2019, Wu & Rastinejad 2017). Endogenous or synthetic agonists and antagonists remain to be identified in this family, but a path forward to successful drug discovery and approval based on their ligand-binding cavities has recently opened (Fallah *et al.* 2022, Keam 2022).

## Declaration of interest

The author is a founder and consultant for Flare Therapeutics.

## Funding

This work did not receive any specific grant from any funding agency in the public, commercial, or not-for-profit sector.
